# CLAST: CUDA implemented large-scale alignment search tool

**DOI:** 10.1186/s12859-014-0406-y

**Published:** 2014-12-11

**Authors:** Masahiro Yano, Hiroshi Mori, Yutaka Akiyama, Takuji Yamada, Ken Kurokawa

**Affiliations:** Department of Biological Information, Graduate School of Bioscience and Biotechnology, Tokyo Institute of Technology, 2-12-1 M6-3, Ookayama, Meguro-ku, Tokyo 152-8550 Japan; Department of Computer Science, Graduate School of Information Science and Engineering, Tokyo Institute of Technology, 2-12-1 W8-76, Ookayama, Meguro-ku, Tokyo 152-8550 Japan; Earth-Life Science Institute, Tokyo Institute of Technology, 2-12-1 E3-10, Ookayama, Meguro-ku, Tokyo 152-8550 Japan

**Keywords:** GPU, CUDA, Metagenomics, Microbial community, Sequence similarity search tool

## Abstract

**Background:**

Metagenomics is a powerful methodology to study microbial communities, but it is highly dependent on nucleotide sequence similarity searching against sequence databases. Metagenomic analyses with next-generation sequencing technologies produce enormous numbers of reads from microbial communities, and many reads are derived from microbes whose genomes have not yet been sequenced, limiting the usefulness of existing sequence similarity search tools. Therefore, there is a clear need for a sequence similarity search tool that can rapidly detect weak similarity in large datasets.

**Results:**

We developed a tool, which we named CLAST (CUDA implemented large-scale alignment search tool), that enables analyses of millions of reads and thousands of reference genome sequences, and runs on NVIDIA Fermi architecture graphics processing units. CLAST has four main advantages over existing alignment tools. First, CLAST was capable of identifying sequence similarities ~80.8 times faster than BLAST and 9.6 times faster than BLAT. Second, CLAST executes global alignment as the default (local alignment is also an option), enabling CLAST to assign reads to taxonomic and functional groups based on evolutionarily distant nucleotide sequences with high accuracy. Third, CLAST does not need a preprocessed sequence database like Burrows–Wheeler Transform-based tools, and this enables CLAST to incorporate large, frequently updated sequence databases. Fourth, CLAST requires <2 GB of main memory, making it possible to run CLAST on a standard desktop computer or server node.

**Conclusions:**

CLAST achieved very high speed (similar to the Burrows–Wheeler Transform-based Bowtie 2 for long reads) and sensitivity (equal to BLAST, BLAT, and FR-HIT) without the need for extensive database preprocessing or a specialized computing platform. Our results demonstrate that CLAST has the potential to be one of the most powerful and realistic approaches to analyze the massive amount of sequence data from next-generation sequencing technologies.

**Electronic supplementary material:**

The online version of this article (doi:10.1186/s12859-014-0406-y) contains supplementary material, which is available to authorized users.

## Background

The rapid development of sequencing technologies has resulted in a flood of new data. For example, a single run of the latest version of the Illumina sequencing system (HiSeq 2500) can produce ~540–600 Gb of sequences with 100-bp read lengths, and can take >11 days [1]. These technologies have made it easier to perform massive sequencing projects such as metagenomic analyses. For example, 3.3 million genes, representing the human gut metagenome, were derived from 124 human fecal samples using next generation sequence technologies [2]. Similarly, the Human Microbiome Project (HMP) produced >8.8 Tb of sequences, representing the normal human metagenome, from 681 samples using the Illumina Genome Analyzer IIx system [3].

Most fundamental metagenomic analyses are highly dependent on sequence alignment tools, such as the Basic Local Alignment Search Tool (BLAST) [4], BLAST-like Alignment Tool (BLAT) [5], and Fragment Recruitment at High Identity with Tolerance (FR-HIT) algorithm [6], to search for nucleotide sequence similarity against sequence databases. The alignment sensitivity of the tool is a crucial factor for metagenomic studies because many of the reads are derived from microbes whose genomes have not yet been sequenced. On the other hand, the search speed is also an important issue because of the increasing amount of data produced from advances in sequencing platforms. For instance, SSEARCH [7], which is an alignment tool based on the Smith–Waterman local alignment algorithm, is too slow to use for massive metagenomic analyses. However, the sensitivity and search speed often have contradictory requirements, and thus most alignment tools used for metagenomic studies sacrifice one of these aspects.

An effective way to accelerate sequence similarity searching while maintaining sufficient sensitivity is to maximize the degree of parallelism. Use of graphics processing units (GPUs) is a suitable way to parallelize calculations with low financial and computational cost, because GPUs are relatively inexpensive, powerful, and widely used for high-performance computing. Many GPU-based sequence similarity search tools have been developed, such as CUDASW++2.0, GPU-BLAST, GHOSTM, G-BLASTN, MUMmerGPU, and SARUMAN [8-13]. However, none of these GPU-based tools is suitable for metagenomic analyses because they cannot detect weak similarity of numerous query nucleotide sequences against reference genomes at reasonably high speed (Additional file 1).

Here, we developed a nucleotide sequence similarity search tool CLAST (CUDA implemented large-scale alignment search tool) that can rapidly detect weak sequence similarity with both short and long query read lengths. CLAST uses GPUs and searches with both global and local alignment algorithms. Global alignment facilitates taxonomic and functional assignment of metagenomic reads, and local alignment is useful in motif searching. Furthermore, we implemented a novel algorithm to construct the q-gram index [14], which allows sequence similarity searching with reference data that has not been pre-indexed. This feature minimizes the memory requirements of CLAST, and allows the use of large and frequently updated reference databases. CLAST was optimized for both the NVIDIA Fermi and more recent Kepler GPU architectures.

## Implementation

### Method of detecting similar regions

**Figure 1:**
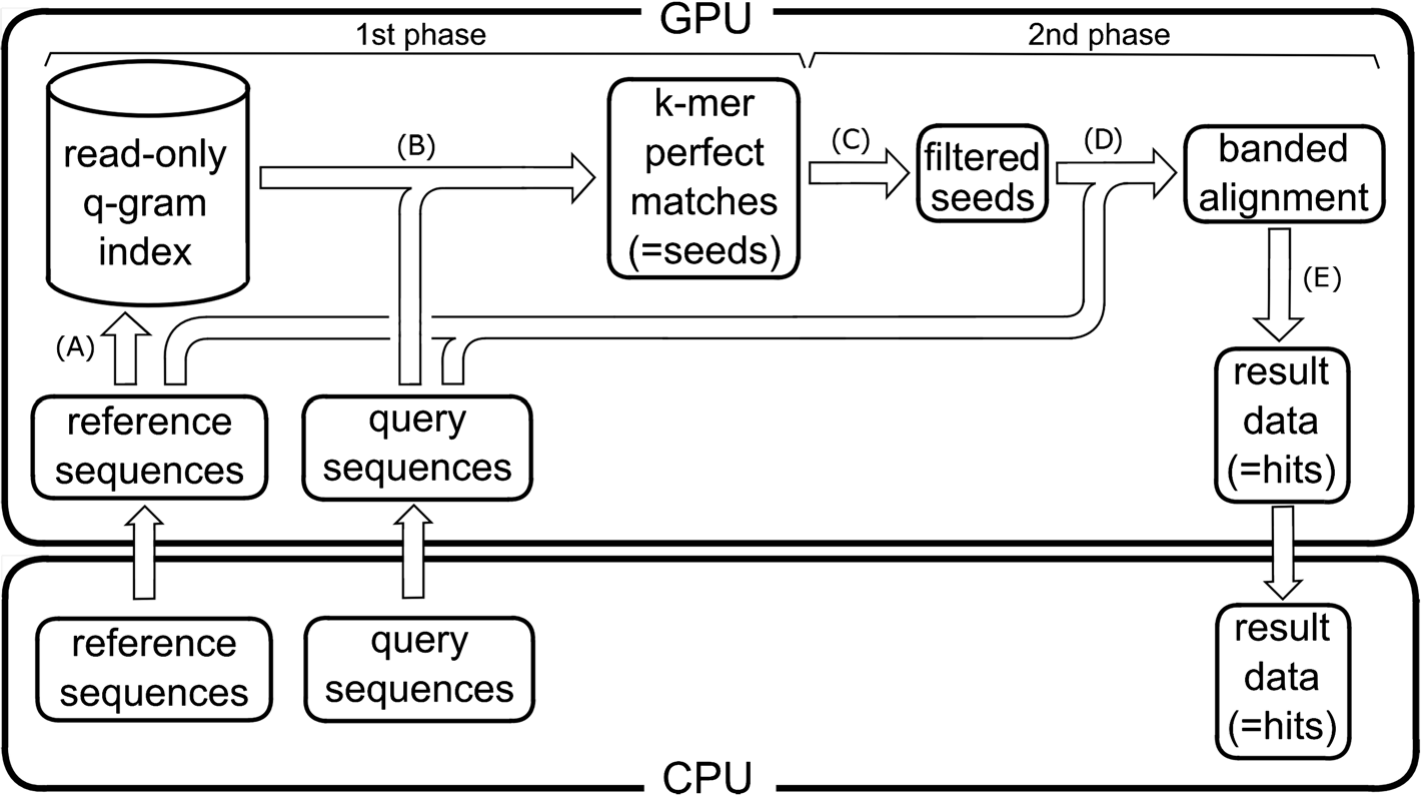
**Overview of the CLAST search processing phases. (A)** A read-only q-gram index was generated from reference genome sequences using a novel algorithm for parallel architecture (Figure 3). **(B)** The query sequences were searched against the read-only q-gram index. **(C)** Seeds were filtered to reduce calculation time (Figure 4). **(D)** The seed sequences were aligned to the reference genome sequences (Figure 2). **(E)** Results were filtered according to E-value and alignment length.

CLAST identifies similar regions between query and reference genome sequences by two phases of processing (Figure 1). In the first phase, CLAST identifies “seeds”, which are regions of reference genome sequences that exactly match query sequences. In the second phase, CLAST executes banded global or local alignment around these seeds [15].

In the first phase, CLAST creates *k*-mers along the reference genome sequence with a sliding window of *k* bases with a step of *p* bases (*p* and *k* are user-adjusted parameters). Next, CLAST constructs a read-only q-gram index from the *k*-mers of the reference sequence that dramatically accelerates similarity searching (the algorithm to create the read-only q-gram index is described below). Finally, CLAST creates seeds by referring *k*-mers across the query sequences to the q-gram index with a sliding window of *k* bases and a step of 1 base.

**Figure 2:**
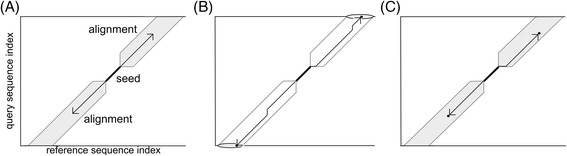
**Banded global and local alignment. (A)** The gray area denotes the region of alignment in this banded alignment. Sequences were aligned from the edges of the seed in both the global and local modes. Sequence comparison ended at the maximal alignment score in gray area in the global alignment **(B)** and local alignment **(C)**.

In the second phase, CLAST performs banded alignments (Figure 2A) for each seed to create “hit” with an identity, a similarity score, and an E-value (Additional file 2). To reduce the alignment calculation time, CLAST only selects seeds that are adjacent to other seeds (within a seed cluster; detail of the algorithm is described below). This process dramatically reduces the calculation time and cost of the subsequent global (default; Figure 2B) and local (optional; Figure 2C) alignments. In the alignments within each seed cluster, CLAST detects similar regions, and calculates identities, and similarity scores. Additional file 3 describes the user-defined parameters by which CLAST controls workflow, such as E-value [16] threshold, *k,* and *p*.

### General algorithm for creating the read-only q-gram index in a parallel architecture

**Figure 3:**
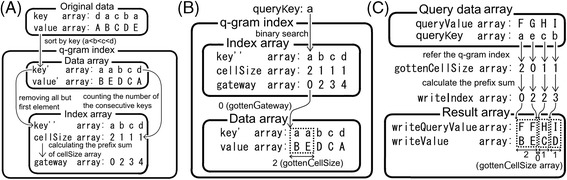
**General algorithm for creating the read-only q-gram index in a parallel architecture. (A)** The parallel algorithm to create a read-only q-gram index. **(B)** The algorithm to obtain a corresponding value stored in the read-only q-gram index of a queryKey. **(C)** The parallel algorithm to obtain the corresponding values stored in the read-only q-gram index of many queryKey.

We generated a new algorithm for creating and referencing a read-only q-gram index that was optimized for parallel architectures, such as GPUs (Figure 3). This q-gram index is not implemented in a hash table, and consequently the memory requirements do not depend on the variety of hash keys but rather on the number of elements that the q-gram index contains. Therefore, in this q-gram index, the key values can be as large as the limit of variables (for instance, if a key is a 64-bit integer, it can be from −2^63^ to 2^63^ − 1). This design enabled *k* to have a value up to 31 in the CLAST q-gram index.

Firstly, we describe the procedure for constructing the read-only q-gram index that consists of data and index arrays from the original data that consisted of keys and values (independent of each other) of each element in the original data (Figure 3A). The data array consists of a sorted key array and a value array sorted by keys. The index array consists of the sorted non-redundant key array (redundant key elements removed), a cellSize array (the redundancy number of each key element), and a gateway array (exclusive prefix summation of the cellSize array).

Secondly, we describe the way to obtain the corresponding values stored in the read-only q-gram index of a queryKey (Figure 3B). A binary search of the sorted non-redundant key array provides the corresponding cellSize and gateway arrays of the queryKey (referred to as gottenCellSize and gottenGateway). The queryKey corresponds to the elements located from the gottenGateway to [gottenGateway + gottenCellSize −1] in the value array (as writeValues).

Finally, we describe the way to write the queryValue array and its corresponding values stored in the read-only q-gram index (Figure 3C). Referencing the read-only q-gram index values by the queryKey (usually generated from queryValue) array creates the gottenCellSize and writeValue arrays. To assign each queryValue and corresponding writeValue to a result array, a writeIndex array was computed by an exclusive prefix sum operation of the gottenCellSize array. The queryValue and corresponding writeValues are written as the location from the writeIndex to the [writeIndex + gottenCellSize −1] in the result array.

In CLAST, the key and value arrays of the original data are the *k*-mer and *k*-mer position hash keys, respectively. In addition to the general algorithm for creating the q-gram index, CLAST overwrites elements of the cellSize array that are larger than the repeat threshold (user-adjustable parameter) with zero to minimize uninformative sequence search seeds. In CLAST, the queryKey and queryValue arrays are the hash key and position information of each *k*-mer of the query sequences, respectively. Therefore, in CLAST, the result array indicates the correspondence of the *k*-mer position in the reference and query sequences and thus is the seed array.

### Algorithm to reduce the number of seeds

**Figure 4:**
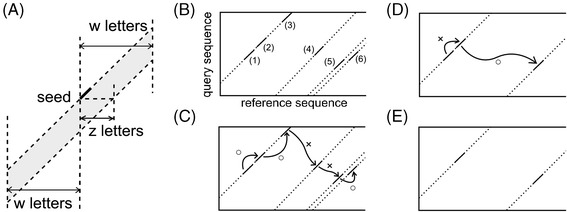
**Algorithm to reduce the number of seeds. (A)** The gray area represents the “surrounding area” of each seed. **(B)** An example of seeds that is to be reduced. The number of each seed represents the order of seeds that is sorted by its position. **(C)** The first algorithm to check the seeds. A balloon means that next seed is in surrounding area, and a x-mark means not. CLAST removes the seeds with x-mark. **(D)** The second algorithm to check the seeds. A x-mark means that next seed is in surrounding area, and a baloon means not. CLAST removes the seeds with x-mark. **(E)** The seeds that remains in this example. The seeds are isolated, there is no seeds in surrounding area.

We defined “surrounding area” of each seed as the same query sequence, same reference sequence, within *z* (user-adjustable parameter) bases parallel to the diagonal, and within *w* (user-adjustable parameter) bases in the reference sequence direction (Figure 4A). First, CLAST sorts seeds by location in the reference sequence (Figure 4B). Next, CLAST removes seeds the next of those are not in surrounding area (Figure 4C). Then, CLAST removes seeds the next of those are in adjacent (Figure 4D). CLAST has removed isolated seeds and arranges seed clusters into its representative seeds for further steps (Figure 4E).

### Splitting of long reference sequences for alignment

Because of the small working memory in GPUs, CLAST is limited in the length of sequences that it can manipulate. Therefore, reference genome sequences longer than the user-defined limit *L* (*default value is* 64 Mb) must be split into shorter overlapping sequence fragments with a CLAST accessory tool. Because all prokaryotic genomes obtained to date are shorter than the default value of *L*, these reference genome sequences do not need to be fragmented for alignment.

### Other specifications

Each CLAST process uses one GPU, and users can specify the GPU on which CLAST runs. This design allows a GPU queuing system to control CLAST processes.

## Results

### CLAST accuracy evaluation by comparison with the Smith–Waterman algorithm

To measure the search accuracy of CLAST, we compared the output results of BLAST 2.2.25 [4], BLAT 34 [5], and CLAST against that of SSEARCH version 36.3.6 [7] (hereafter referred to as the accuracy test). We chose only BLAST and BLAT in the accuracy test because these two tools are widely used in metagenomic analyses (e.g. MEGAN, which is a commonly used taxonomic and functional assignment tool for metagenomics, uses BLAST results for their taxonomic and functional assignment [17]; MG-RAST, which is a commonly used metagenomic analyses web service, uses BLAT for their sequence similarity analyses [18]). This comparison consisted of six phases. First, we obtained the reference genome sequences of all bacteria and archaea in the National Center for Biotechnology Information (NCBI) RefSeq Genome database (October 2011, 4.3 GB, 2,314 sequences) [19] that were completely sequenced and had full taxonomic information. Second, we created two query sets (100-base test; 10,000 reads of 100 bases as simulated-Illumina reads, 800-base test; 10,000 reads of 800 bases as simulated-454 reads) by randomly retrieving 100-base and 800-base sequence fragments from the 2,314 reference genome sequences. Thirdly, these query sets were searched against the reference genome sequences using SSEARCH, BLAST, BLAT, and CLAST. Fourthly, we removed hits from the results for each alignment tool if the assigned regions and query were from the same reference genome sequence. This step makes the result equivalent to a search for the query sequence in the reference sequence database without the original query genome sequence. Fifthly, we selected the best non-self hits from the result of each tools with the scoring criteria dependent on the alignment tool. Then sixthly, BLAST, BLAT, and CLAST were considered to accurately find a hit when they reported the same hit and alignment position as SSEARCH. This accuracy test was performed on a desktop computer with an Intel Xeon X5670 6 core 2.93 GHz CPU, 48 GB main memory, and two NVIDIA Tesla C2050 GPUs.

### Results of comparison of search accuracy

**Figure 5:**
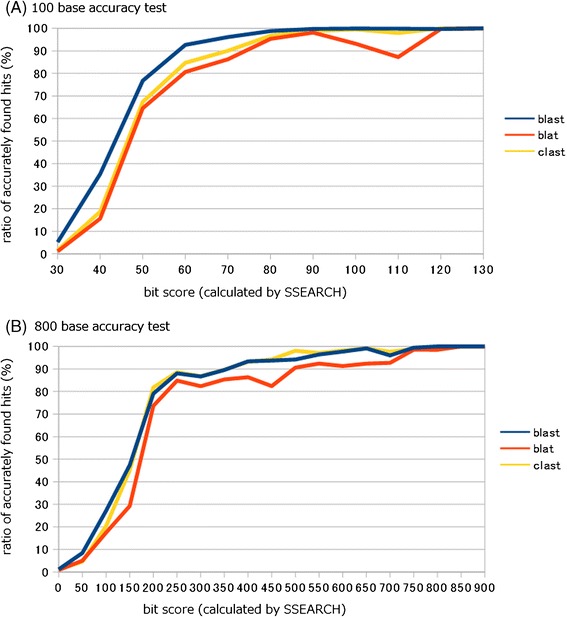
**Result of each accuracy test.** Both of the graph represent the results of simulated metagenomic analysis test. Horizontal axis represents bit score calculated by SSEARCH, and vertical axis represents ratio of accurately found hits. **(A)** Results of 100 base accuracy test. **(B)** Results of 800 base accuracy test.

In both the 100- and 800-base accuracy tests, the search accuracy of CLAST was comparable to that of BLAST, both when bit scores were >90 (100-base test) or 200 (800-base test) and when bit scores were <90 (100-base test) or 200 (800-base test). The search accuracy of CLAST was greater than that of BLAT in nearly all cases (Figure 5 and Additional file 4).

### Evaluating speed, sensitivity, and accuracy of taxonomic assignments

Massive metagenomic analyses generally depend on the alignment for each read against reference genomes to assign taxonomy for the read. Therefore, we designed a simulated metagenomic analysis test to evaluate the sensitivity and accuracy of the taxonomic assignments as well as calculation time.

**Figure 6:**
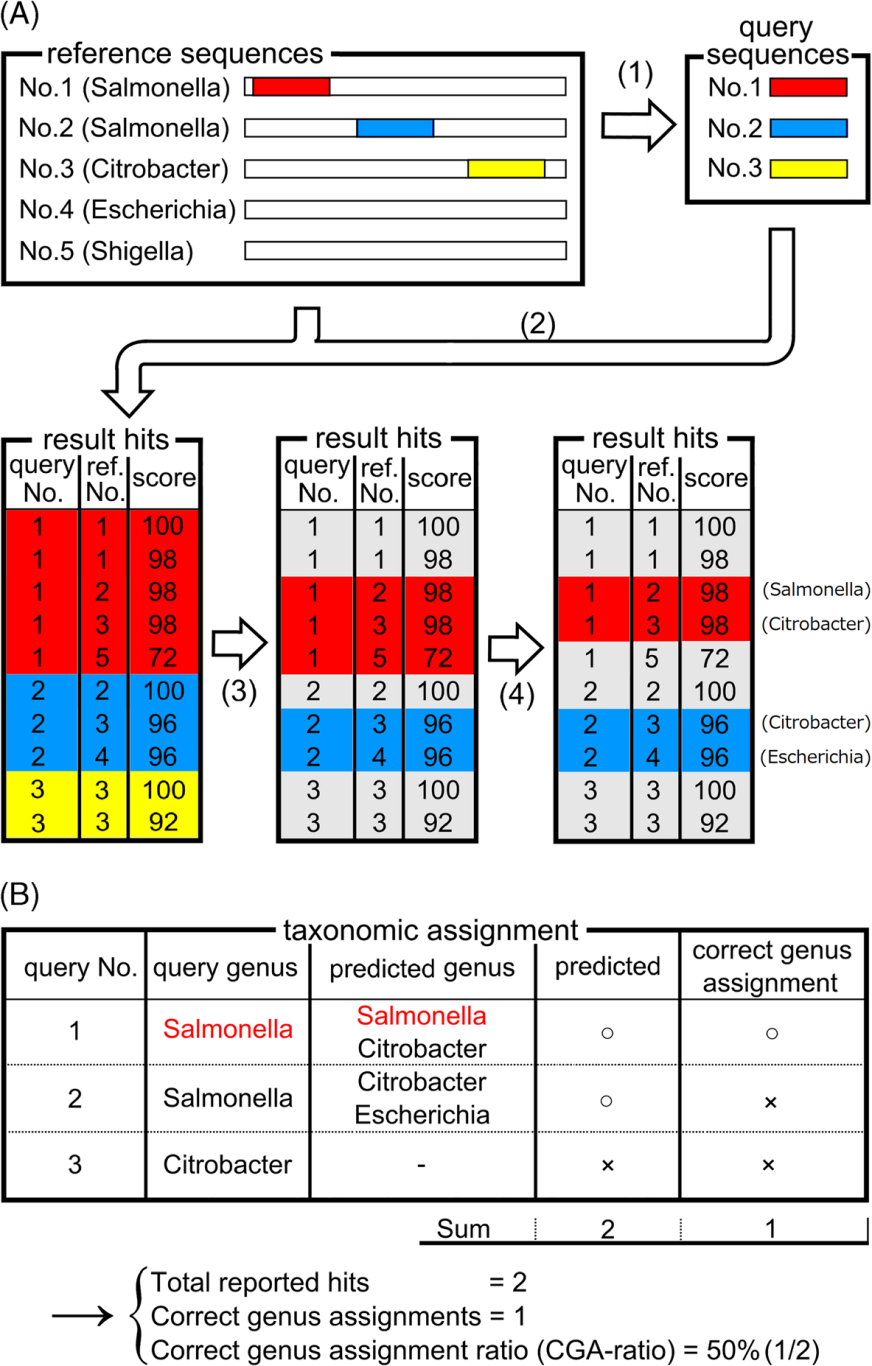
**Comparison of the search accuracy of different alignment tools. (A)** Taxonomic assignment of the query sequences in the simulated metagenomic analysis test was performed in the following steps: 1: Query sequences were generated by randomly selecting short fragments from reference genome sequences. 2: Sequence similarities were calculated between the query and reference genome sequences. 3: If a query matched to the original reference sequence, it was deleted from the results. 4: The best non-self hits were selected for taxonomic assignment. **(B)** Whether the result of taxonomic assignment were correct or not were assessed based on the taxonomic databases.

The simulated metagenomic analysis test consisted of six phases. First, we created two query sets (100,000 reads of 100 or 800 bases) from 2,314 reference genome sequences as in the accuracy test. Second, we searched for similar regions between each query sequence and the reference genome sequences. Third, we removed hits from the results if the assigned region and query were from the same reference genome sequence. Fourth, we selected the best non-self hits from the result of each tool, as in the previous accuracy test. Fifth, taxonomic assignment of the query sequences was performed using taxonomy of the best non-self hits (Figure 6A). Finally, we counted the number of query sequences that had similar regions in any reference genome (total reported hits) and the number of queries that were correctly taxonomically assigned (correct genus assignments) (Figure 6B). We compared the number of total reported hits, the number of correct genus assignments, and the correct genus assigned ratio (CGA ratio; number of correct genus assignments/number of total reported hits) among the tools tested. Total reported hits is a measure of the alignment tool sensitivity, and CGA ratio is a measure of the accuracy of taxonomic assignment. Since more sensitive similarity search tools will detect weaker similarity, and consequently will have a greater number of total reported hits, these tools are more useful for motif searching (Figure 6).

In the simulated metagenomic analysis test, we compared CLAST with other similar tools, namely BLAST 2.2.25, BLAT 34, FR-HIT v0.6, Burrows–Wheeler Aligner (BWA)/BWA-SW 0.5.9, Bowtie 2 2.0.4, and G-BLASTN 1.1, which depends on BLAST 2.2.28+ [4-6,20-22]. G-BLASTN was separately compared with CLAST because G-BLASTN was designed for the NVIDIA Kepler architecture GPU. The default command line options were used for each alignment tool tested (Additional file 5). BWA/BWA-SW 0.5.9, Bowtie 2 2.0.4, and BLAT 34 cannot handle databases larger than 4 GB [23-25]. Therefore, we separated the reference genome sequences into three sets for testing these programs. Similarity search results from the three sets were merged for comparison with the results from BLAST, CLAST, and FR-HIT. The best non-self hits were selected using CIGAR code and MD tag (BWA), E-value (FR-HIT), and alignment score (Bowtie 2, BWA-SW, BLAST, BLAT, and CLAST). The simulated metagenomic analysis test (except for G-BLASTN) was performed on the same desktop computer as the accuracy test.

### Results of comparison of calculation time between CLAST and other tools

In the 100-base test, Bowtie 2 (global mode) was the fastest tool, followed by Bowtie 2 (local mode), BWA, CLAST (global mode), CLAST (local mode), BLAT, FR-HIT (both global and local modes), and BLAST. CLAST (global mode) was 72.6 times faster than BLAST. CLAST (local mode) speed was comparable to CLAST (global mode) and 2.35 times faster than BLAT.

**Figure 7:**
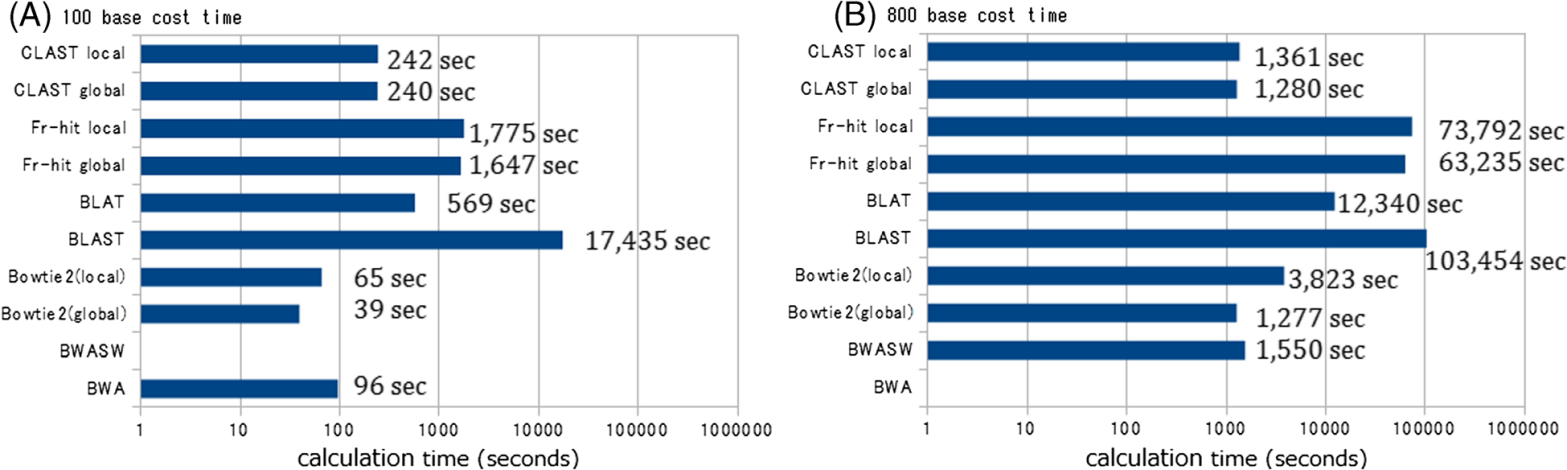
**Search calculation time of each simulated metagenomic analysis test.** The time for each tool to search 100,000 query reads against 2,314 reference genome sequences. Horizontal axis represents calculation time. **(A)** Results of 100 base test. **(B)** Results of 800 base test.

In the 800-base test, Bowtie 2 (global mode) and CLAST (both global and local modes) were the fastest tools. The calculation time of CLAST was comparable to Bowtie 2 (global mode) and faster than BWA-SW (Figure 7). CLAST (global mode) was 9.64 and 80.8 times faster than BLAT and BLAST, respectively.

### Results of comparison of similarity search sensitivity and accuracy of taxonomic assignment

**Figure 8:**
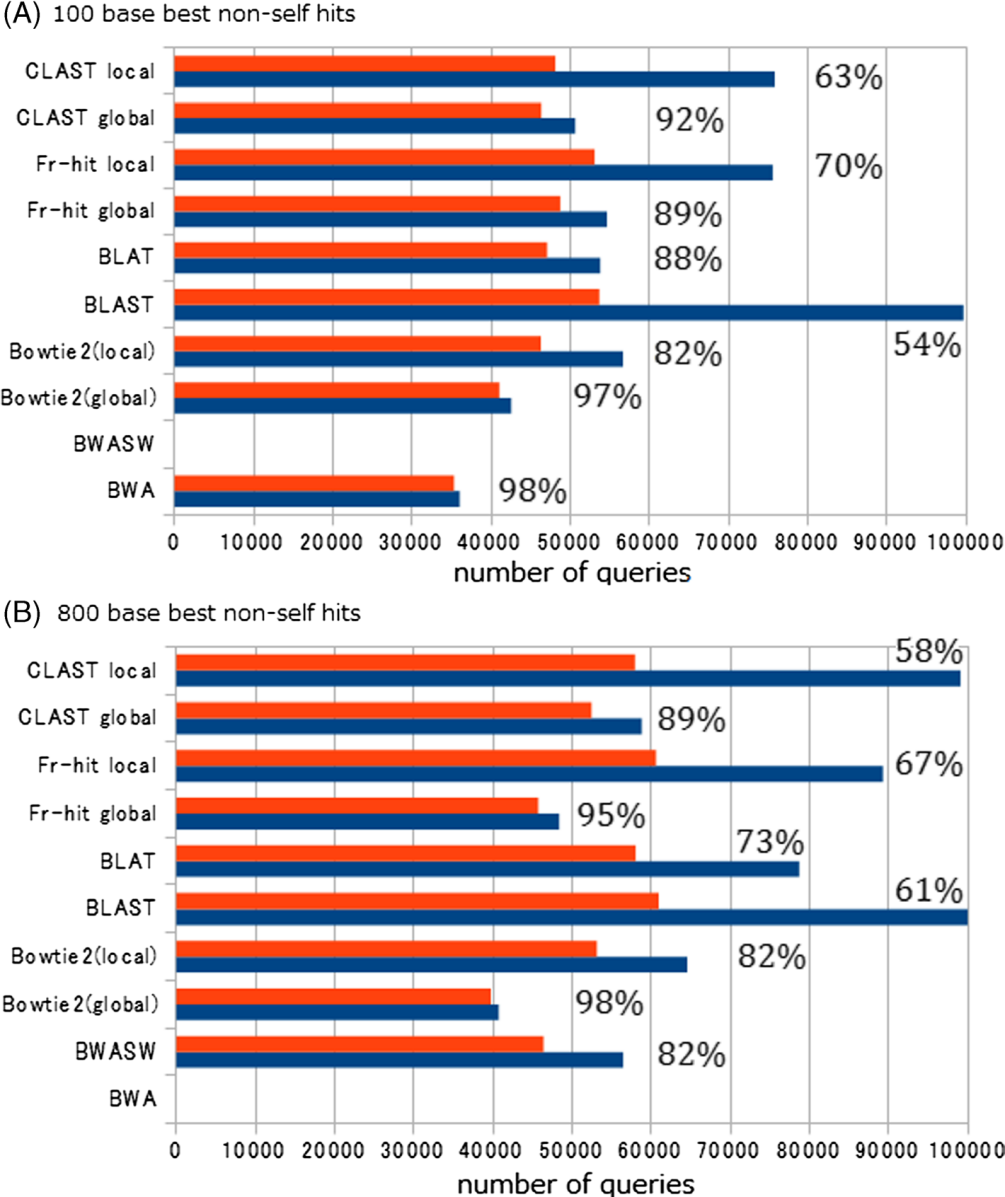
**Results of the simulated metagenomic analysis test.** Blue: Number of query reads that had at least one similar sequence in the database (total reported hits). Red: Number of query reads with correct taxonomic assignment (correct genus assignments). Percentages are the CGA ratio (correct genus assignments/total reported hits × 100). Horizontal axis represents number of queries. **(A)** Results of 100 base test. **(B)** Results of 800 base test.

In the 100-base test, the highest number of total reported hits (highest sensitivity) was obtained with BLAST (Figure 8 and Additional file 6), followed by FR-HIT (local mode), CLAST (local mode), and the remaining tools. In the 800-base test, the highest number of total reported hits was also obtained with BLAST; however, CLAST (local mode) obtained nearly as many total reported hits, whereas the other tools obtained lower numbers. These results indicate that, for both read lengths, BLAST, FR-HIT (local mode), and CLAST (local mode) achieved high sensitivity, and that CLAST (local mode) is sensitive enough to map metagenomic reads to reference genome sequences.

**Figure 9:**
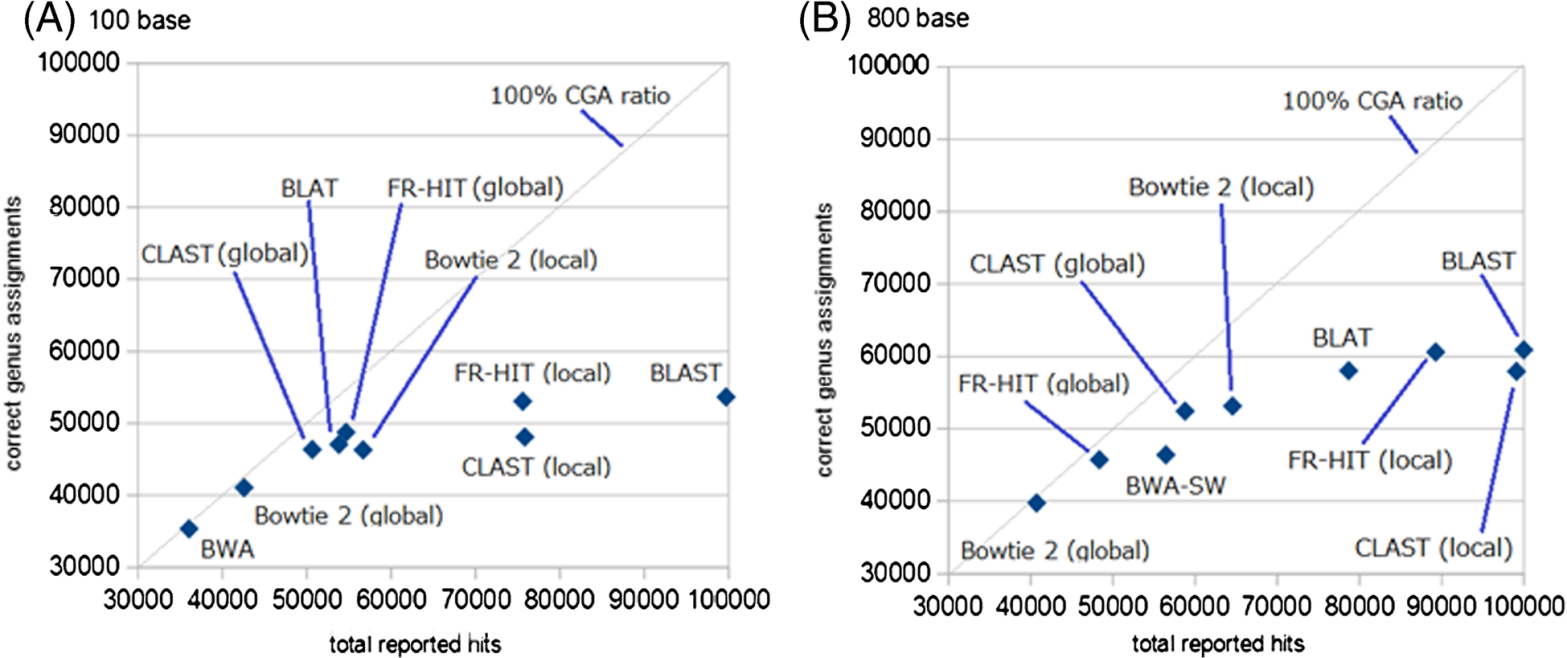
**Relationships of sensitivity (total reported hits) and specificity (correct genus assignments) for each software both of the 100 base test and the 800 base test.** Each point represents the result of simulated metagenomic analysis of BLAST, BLAT, CLAST (both global and local mode), FR-HIT (both global and local mode), BWA, BWA-SW, Bowtie 2 (both global and local mode). The gray slanting line of each graph represents 100 % CGA ratio. All points cannot be above the gray line. Horizontal axis represents total reported hits, and vertical axis represents correct genus assignments. **(A)** Results of 100 base test. **(B)** Results of 800 base test.

**Figure 10:**
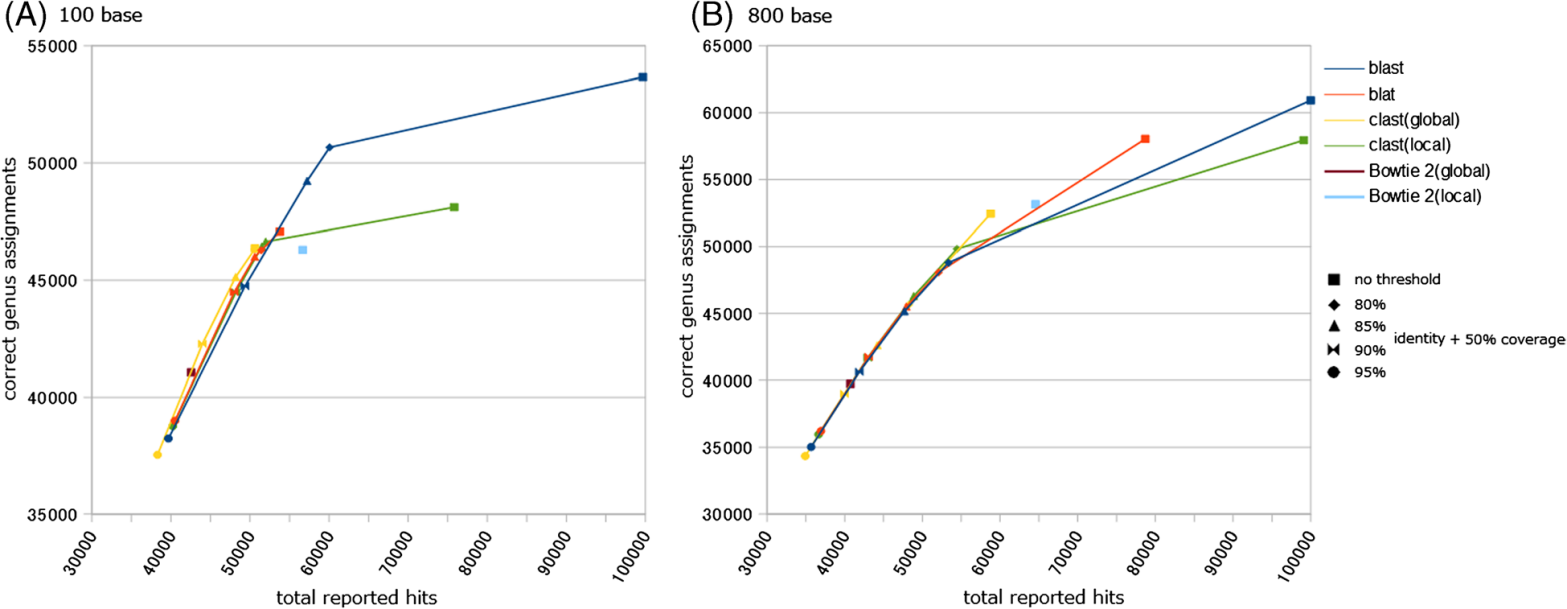
**Relationships between sensitivity and specificity of BLAST, BLAT, and CLAST by changing the identity threshold both of the 100 base test and the 800 base test.** Each curve represents the results of simulated metagenomic analysis of BLAST, BLAT, and CLAST (both global and local modes) under several thresholds. Each curve consists of the 5 points, indicating the results of simulated metagenomic analysis with 5 different thresholds. One point is the result that was not filtered by any identity and coverage thresholds (same with the point in Figure 9), and the others are based on the results that were filtered by an identity threshold and a coverage threshold. The identity thresholds were 95%, 90%, 85%, and 80%. The coverage threshold was unified to 50%. In all curves, high identity thresholds represent small numbers of total reported hits and correct genus assignments (Additional file 7). The points of Bowtie 2 results (both global and local modes) that were not filtered by any identity and coverage thresholds (same with the points in Figure 9) are also plotted to be able to compare with the curves of other tools. Horizontal axis represents total reported hits, and vertical axis represents correct genus assignments. **(A)** Results of 100 base test. **(B)** Results of 800 base test.

For both read lengths, Bowtie 2 (global mode), CLAST (global mode), and FR-HIT (global mode) achieved higher CGA ratios (greater accuracy) than the other tools. In the 100-base test, BWA and Bowtie 2 (global mode) achieved very high CGA ratios (98 and 96%, respectively), but these algorithms produced fewer total reported hits than the other tools. In the 800-base test, the number of total reported hits from BWA-SW was 0.96 and 1.16 times greater than from CLAST (global mode) and FR-HIT (global mode). However, the CGA ratio of BWA-SW was much lower than that of FR-HIT (global mode) and CLAST (global mode) (Figure 8B) because the number of incorrect genus assignments of BWA-SW was 1.58 and 3.79 times greater than those of CLAST (global mode) and FR-HIT (global mode), respectively (Additional file 6). Similarly, the number of total reported hits of Bowtie 2 (local mode) was 1.10 and 1.33 times greater than those of CLAST (global mode) and FR-HIT (global mode), but the number of incorrect genus assignments of Bowtie 2 (local mode) was 1.80 and 4.31 times greater than those of CLAST (global mode) and FR-HIT (global mode) (Additional file 6). These results indicate that global alignment is useful for the purpose of taxonomic assignment. BWA, Bowtie 2 (global mode), FR-HIT (global mode), and CLAST (global mode) are able to assign reads to taxonomic groups with reasonably high accuracy. Especially among these four tools, CLAST (global mode) and FR-HIT (global mode) achieved not only high accuracy of taxonomic assignment but also moderate search sensitivity (Figure 9). Accuracy of taxonomic assignment of CLAST (global mode) and FR-HIT (global mode) excelled those of Bowtie 2 (local mode) and BWA-SW, and search sensitivity of CLAST (global mode) and FR-HIT (global mode) excelled those of Bowtie 2 (global mode) and BWA. In addition, by changing the identity threshold and the coverage threshold, the relationships between total reported hits and correct genus assignment of BLAST, BLAT, and CLAST (both global and local modes) were shown as curves (Figure 10 and Additional file 7). Although, the curves of the all tools were resembled each other, the curve of CLAST (global mode) was slightly higher than that of other tools in the 100 base test. The point of Bowtie 2 (global mode) was near to that of CLAST (global mode) with 90% identity threshold in both tests. The point of Bowtie 2 (local mode) achieved lower CGA ratio than the curves of BLAST, BLAT, and CLAST (global mode) in the 100 base test, and was near to the curve of BLAT in the 800 base test.

### Calculation time using multiple GPUs

We ran CLAST on one, two, and eight GPUs with actual metagenomic reads to investigate the effect of GPU number on the calculation time. The reference genome sequences were the same as that used in the simulated metagenomic analysis test. The query sequences, which are the Illumina Genome Analyzer IIx reads from a human gut microbial community, were obtained from Qin *et al.* [2] (NCBI SRA accession number ERR011343; 75 bp, 21,739,219 reads). For this test, we used a 4-node GPU server. Each of the node had an Intel Xeon X5690 6 core 3.47 GHz CPU, 64 GB main memory, and two NVIDIA Tesla C2075 GPUs.

### Results of calculation time using multiple GPUs

**Figure 11:**
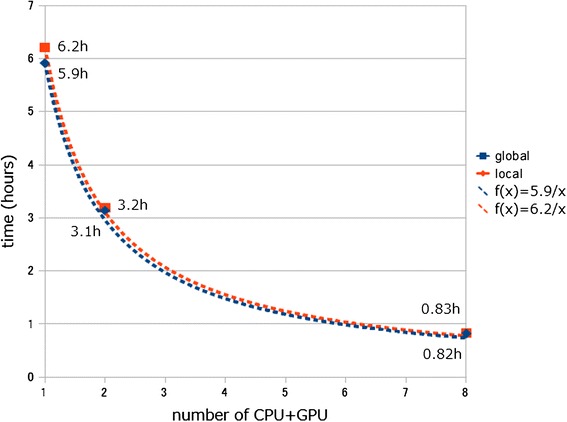
**Calculation time of CLAST with real metagenomic reads.**

Total similarity search-calculation time for CLAST with the real metagenomic reads showed a linear inverse relationship with GPU number (Figure 11). With only one GPU, the calculation times of CLAST were 355 min (global mode) and 373 min (local mode) (Figure 11). With two GPUs, calculation times of CLAST were 188 min (global mode) and 192 min (local mode), and with eight GPUs, calculation times of CLAST were 49 min (global mode) and 50 min (local mode). This result indicates that CLAST function can be greatly accelerated by using multiple GPUs.

### Comparison with G-BLASTN

In addition to comparison of CLAST with CPU-based tools, we compared the speed, sensitivity, and accuracy of CLAST taxonomic assignments to those of G-BLASTN (BLAST algorithm optimized for Kepler architecture GPU computing). The dataset and the analysis pipeline for comparison with G-BLASTN were the same as those of the simulated metagenomic analysis test. We used a workstation with two Intel Xeon E5-2687 W 8 core 3.10 GHz CPUs, 62.9 GB main memory, and two NVIDIA Tesla K20m GPUs (hereafter referred to as the two K20 machine). If the CLAST algorithm achieves the same speed as that of G-BLASTN, G-BLASTN (default settings) would be approximately two times as fast as CLAST (default settings) on the two K20 machine because G-BLASTN automatically uses all available GPUs, and one CLAST process uses only the one specified GPU. We compared CLAST to G-BLASTN run in the megablast mode (designed to identify only similar sequences) and blastn modes (command line parameters are -use_gpu true -outfmt 6 -task megablast and -use_gpu true -outfmt 6 -task blastn).

### Results of comparison with G-BLASTN

In the simulated metagenomic analysis test, the G-BLASTN (blastn mode) analysis took 15,970 s when the query length was 100 bases, and 136,560 s when the query length was 800 bases, on the two K20 machines. On the other hand, CLAST took 210 s (global mode) and 215 s (local mode) for the 100-base query length, and 1,248 s (global mode) and 1,352 s (local mode) for the 800-base query length in the same GPU architecture. In other words, CLAST was 150–200 times faster than G-BLASTN (blastn mode). Furthermore, G-BLASTN (megablast mode) took 199 s when query length was 100 bases, and 724 s when query length was 800 bases. Thus, CLAST was 1.07–1.85 times faster than G-BLASTN (megablast mode). These results suggest that CLAST is much faster than G-BLASTN (blastn mode) and slightly faster than G-BLASTN (megablast mode).

The total reported hits and correct genus assignments of G-BLASTN (blastn mode) were 99,841 and 56,151, respectively (CGA ratio: 58%), when the query length was 100 bases. The total reported hits and correct genus assignments of G-BLASTN (blastn mode) were 100,000 and 62,728, respectively (CGA ratio: 63%), when the query length was 800 bases. Thus, G-BLASTN (blastn mode) performed similarly to BLAST in the simulated metagenomic analysis test. This result suggests that CLAST (local mode) can detect as much information as G-BLASTN (blastn mode) when the query length is 800 bases.

The total reported hits and correct genus assignments of G-BLASTN (megablast mode) were 46,720 and 42,664, respectively (CGA ratio: 91%), when the query length was 100 bases. The total reported hits and correct genus assignments of G-BLASTN (megablast mode) were 65,108 and 52,754, respectively (CGA ratio: 81%), when the query length was 800 bases. Thus, G-BLASTN (megablast mode) was similar to Bowtie 2 (local mode) in the simulated metagenomic analysis test. This result showed that the accuracy of taxonomic assignments of CLAST (global mode) is greater than that of G-BLASTN (megablast mode) and that the sensitivity of CLAST (local mode) is greater than that of G-BLASTN (megablast mode).

## Discussion

**Figure 12:**
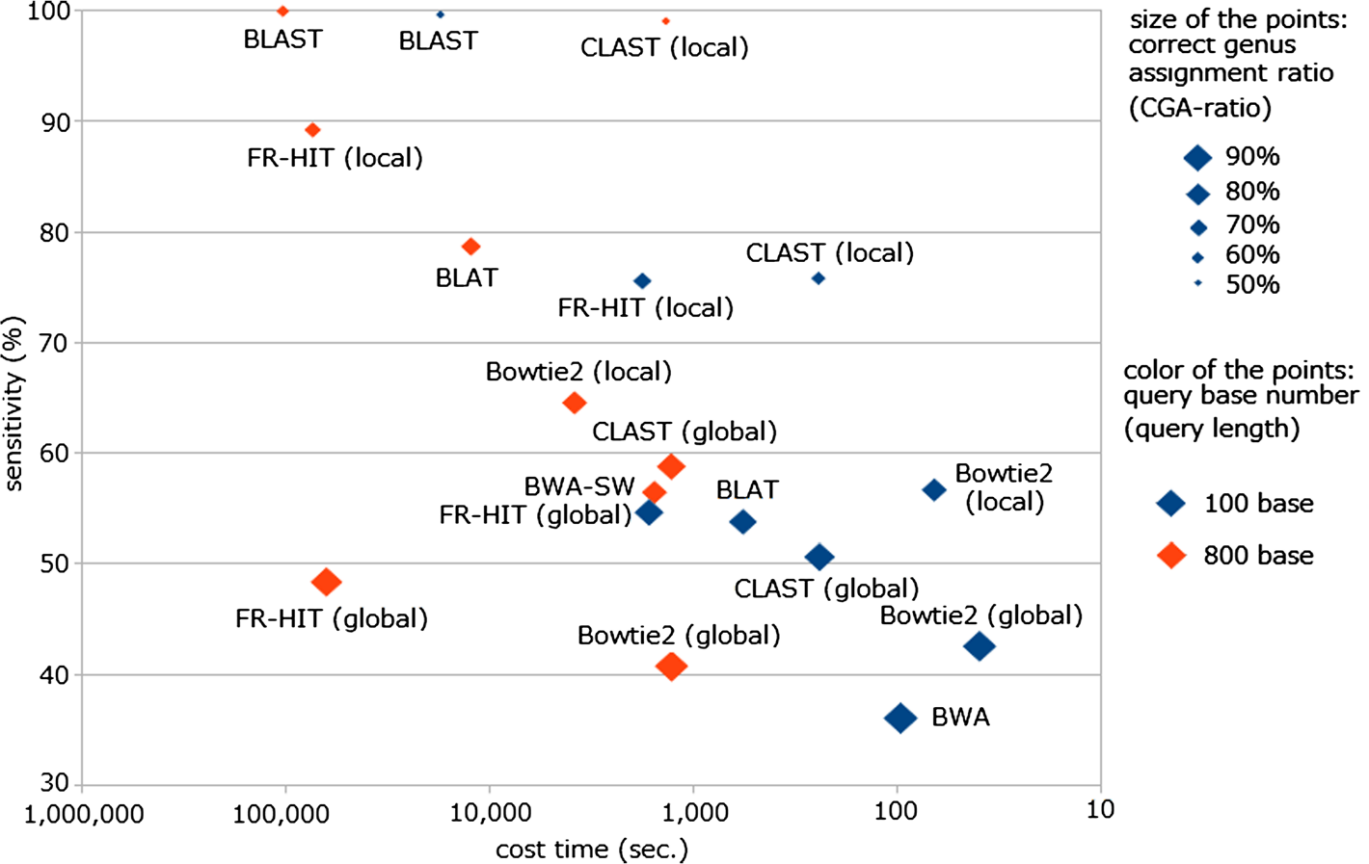
**Scatter diagram of sensitivity versus time use.**

As mentioned above, both high speed and accuracy of similarity searching are necessary for analyses of the large number of reads, often from uncharacterized microbes, derived from high-throughput several metagenomic sequencing projects. CLAST is an ultrafast and sensitive similarity-searching tool that is optimized for massive metagenomic analyses with next-generation sequencing technologies (Figure 12). Here we demonstrated the excellent performance of the CLAST tool in terms of both computation speed and sensitivity. The high speed of CLAST largely comes from the use of GPUs, which are relatively inexpensive, powerful, and widely used to accelerate high-performance computing. The sensitivity of CLAST largely comes from the use of banded dynamic alignment as a programming algorithm for seed extension. Moreover, the speed and sensitivity of CLAST can be improved by specifying longer or shorter *k* values, respectively. However, CLAST may not be appropriate for users who are not familiar with GPU computing, and because CLAST is designed for metagenomic analyses, other tools are more suitable in other situations. For example, BWA or Bowtie 2 are more appropriate for data analyses in genome resequencing projects for which there is a reference genome, and thus a higher-sensitivity tool is not required (Figure 12).

In addition to its speed and sensitivity, the dynamic programming of CLAST also allows both global and local alignments. Global alignment is useful for assigning taxonomic assignment of metagenomic reads because global alignments can evaluates similarity of large regions of the query and reference genome sequences (Figure 8). Our results demonstrate that CLAST (global mode) produces highly accurate taxonomic assignments, similar to several other global alignment tools. CLAST (global mode) also achieved relatively high sensitivity, which is higher than those of BWA and Bowtie 2 (global mode) (Figure 8). On the other hand, local alignment is useful for motif searching, which is often used in functional metagenomics [26], because it can identify partial sequence identity between reads and reference genome sequences. The unique ability of CLAST to perform both local and global alignment greatly enhances its usefulness in metagenomics analyses (see Additional file 8).

Large-scale metagenomic analyses often require use of very large and frequently updated reference databases. CLAST is exceptionally suited for these analyses because it requires minimal database preprocessing for larger genome sequences and does not have a database size limitation. In addition, the maximum memory usage of CLAST is independent of the size of the reference genome sequences in the database.

Although some other alignment tools are able to perform large-scale similarity searches, they tend to require more database preprocessing and memory usage than CLAST. For example, BLAST requires database preprocessing, and the calculation time of this preprocessing is highly related to the size of database. Although BLAT does not require preprocessing, it cannot utilize databases larger than 4 GB [23]. FR-HIT does not require preprocessing, but its memory usage is typically two or three times larger than the size of the database [6]. Burrows–Wheeler transform-based mapping tools usually also require database preprocessing, but because these mapping tools use block sorting, the preprocessing time is generally incidental. For instance, preprocessing of the human genome database (~3 GB) by Burrows–Wheeler transform-based tools usually takes only a few hours [24,25]. However, the microbial genome sequence database now exceeds 5 GB, and the NCBI non-redundant nucleotide sequence database is now >40 GB, and these databases will continue to grow. Given its unique ability to use these extremely large and rapidly growing databases, CLAST shows great promise as an alignment tool for genomic and metagenomic analyses (see Additional file 8).

CLAST requires ~2 GB of main memory and ~2 GB of VRAM, under default settings, and usual metagenomic analyses. More VRAM may be consumed when numerous outputs are produced compared with the input query and reference sequences, such as in 16S rRNA gene amplicon sequencing analyses, but users can manipulate the VRAM usage by specifying specific parameters. The low memory usage of CLAST makes it a reasonable approach for large-scale metagenomic analyses by researchers who do not have access to specialized large-memory computers. This low memory usage is achieved by dividing both the reference genome sequences and query sequences into smaller units that are loaded stepwise to the main memory. Although CLAST depends on a q-gram index of reference sequences, creation of the read-only q-gram index is also performed by the GPUs and therefore does not add substantially to the running time. This feature is one of the most important and innovative advances of CLAST. In contrast, BLAT and FR-HIT load all reference genome sequence data to the main memory at the same time, thus requiring a larger main memory for larger databases. Indeed, FR-HIT used >13 GB of memory in our simulated metagenomic analysis test.

To further take advantage of parallel-computation power, CLAST can be run by multiple GPUs, dramatically accelerating the homology search. This feature, combined with its low memory usage, makes CLAST appropriate for GPU clusters and supercomputers, which are often equipped with nodes having more than one GPU and less than 100 GB of memory. The source code of the CLAST tool is attached to this paper (Additional file 9).

## Conclusions

The novel high-speed and sensitive sequence similarity search tool CLAST was designed and validated for metagenomic analysis applications. CLAST was capable of identifying sequence similarities ~80.8 times faster than BLAST and 9.6 times faster than BLAT owing to a GPU-based parallelization technique using CUDA computing architecture. To improve the sensitivity of similarity searching for taxonomic assignment and motif searching, CLAST supports both global and local alignment. Furthermore, CLAST does not require extensive database preprocessing, and consequently can be run on a standard desktop computer with NVIDIA GPUs. Taken together, our results demonstrate that CLAST run on a GPU-oriented cluster or supercomputer has the potential to be one of the most powerful and realistic approaches to analyze the massive amount of sequence data from next-generation sequencing technologies.

## Availability and requirements

- **Project name:** CLAST
- **Project home page:**https://github.com/masayano/CLAST
- **Operating system(s):** Platform independent
- **Programming language:** CUDA
- **Other requirements:** NVIDIA Fermi architecture GPU, CUDA 4.0
- **License:** GNU GPL
- **Any restrictions to use by non-academics:** None

## Additional files

Additional file 1
**Features of GPU-based similarity searching tools.** (*) CUDA SW++ calculates 17 giga cell updates per second on a single-GPU GeForce GTX 280 graphics card [8]. Thus, it takes ~4 million seconds for CUDA SW++ to search for similar regions between 1 million 100-base query sequences and 4 billion bases of reference sequences. Therefore, CUDA SW++ is not suitable for analysis of reads from next-generation sequencing technologies. (**) GPU-BLAST is ~3–4 times faster than NCBI BLAST, but it is slower than BLAT [5,9]. (***) Demonstrated in the Results section. Additional file 2
**Information about the similar regions of CLAST hits.**
Additional file 3
**Parameters used in CLAST and its description.**
Additional file 4
**Detailed results of the accuracy test.**
Additional file 5
**Command line parameters for each tool in the simulated metagenomic analysis test.**
Additional file 6
**Detailed results of the simulated metagenomic analysis test.**
Additional file 7
**Detailed results of the simulated metagenomic analysis test with changing threshold values.**
Additional file 8
**Specifications of the tools used in the simulated metagenomic analysis test.** (*) BWA/BWA-SW 0.5.9 was used in this study; however, more recent BWA algorithms (since 0.6.x) can now utilize genomes >4 GB [24]. Additional file 9
**Source code of the CLAST tool.**

## References

[1] **Performance and Specifications for HiSeq 2500/1500** [http://www.illumina.com/systems/hiseq_2500_1500/performance_specifications.ilmn]

[2] Qin J, Li R, Raes J, Arumugam M, Burgdorf KS, Manichanh C, Nielsen T, Pons N, Levenez F, Yamada T, Mende DR, Li J, Xu J, Li S, Li D, Cao J, Wang B, Liang H, Zheng H, Xie Y, Tap J, Lepage P, Bertalan M, Batto J-M, Hansen T, Le Paslier D, Linneberg A, Nielsen HB, Pelletier E, Renault P (2010). A human gut microbial gene catalogue established by metagenomic sequencing. Nature.

[3] The Human Microbiome Project Consortium (2012). A framework for human microbiome research. Nature.

[4] Altschul SF, Gish W, Miller W, Myers EW, Lipman DJ (1990). Basic local alignment search tool. J Mol Biol.

[5] Kent WJ (2002). BLAT–the BLAST-like alignment tool. Genome Res.

[6] Niu B, Zhu Z, Fu L, Wu S, Li W (2011). FR-HIT, a very fast program to recruit metagenomic reads to homologous reference genomes. Bioinformatics.

[7] Pearson WR (1991). Searching protein sequence libraries: comparison of the sensitivity and selectivity of the Smith-Waterman and FASTA algorithms. Genomics.

[8] Liu Y, Schmidt B, Maskell DL (2010). CUDASW++2.0: enhanced Smith-Waterman protein database search on CUDA-enabled GPUs based on SIMT and virtualized SIMD abstractions. BMC Res Notes.

[9] Vouzis PD, Sahinidis NV (2011). GPU-BLAST: using graphics processors to accelerate protein sequence alignment. Bioinformatics.

[10] Suzuki S, Ishida T, Kurokawa K, Akiyama Y (2012). GHOSTM: A GPU-accelerated homology search tool for metagenomics. PLoS One.

[11] Zhao K, Chu X (2014). G-BLASTN: accelerating nucleotide alignment by graphics processors. Bioinformatics.

[12] Schatz MC, Trapnell C, Delcher AL, Varshney A (2007). High-throughput sequence alignment using Graphics Processing Units. BMC Bioinformatics.

[13] Blom J, Jakobi T, Doppmeier D, Jaenicke S, Kalinowski J, Stoye J, Goesmann A (2011). Exact and complete short read alignment to microbial genomes using GPU programming. Bioinformatics.

[14] Jokinen P, Ukkonen E (1991). Two algorithms for approximate string matching in static texts. Lect Notes Comput Sci.

[15] Chao KM, Pearson WR, Miller W (1992). Aligning two sequences within a specified diagonal band. Comput Appl Biosci.

[16] **The Statistics of Sequence Similarity Scores.** [http://www.ncbi.nlm.nih.gov/BLAST/tutorial/Altschul-1.html]

[17] Huson DH, Weber N (2013). Microbial community analysis using MEGAN. Methods Enzymol.

[18] Wilke A, Glass EM, Bartels D, Bischof J, Braithwaite D, D'Souza M, Gerlach W, Harrison T, Keegan K, Matthews H, Kottmann R, Paczian T, Tang W, Trimble WL, Yilmaz P, Wilkening J, Desai N, Meyer F (2013). A metagenomics portal for a democratized sequencing world. Methods Enzymol.

[19] **NCBI RefSeq Genome Database.** [ftp://ftp.ncbi.nih.gov/genomes/Bacteria/]

[20] Li H, Durbin R (2009). Fast and accurate short read alignment with Burrows-Wheeler transform. Bioinformatics.

[21] Li H, Durbin R (2010). Fast and accurate long read alignment with Burrows-Wheeler transform. Bioinformatics.

[22] Langmead B, Salzberg SL (2012). Fast gapped-read alignment with Bowtie 2. Nat Methods.

[23] **BLAT Suite Program Specifications and User Guide.** [http://genome.ucsc.edu/goldenPath/help/blatSpec.html]

[24] **Burrows-Wheeler Aligner.** [http://bio-bwa.sourceforge.net]

[25] **Bowtie2: Manual.** [http://bowtie-bio.sourceforge.net/bowtie2/manual.shtml]

[26] Dinsdale EA, Edwards RA, Hall D, Angly F, Breitbart M, Brulc JM, Furlan M, Desnues C, Haynes M, Li L, McDaniel L, Moran MA, Nelson KE, Nilsson C, Olson R, Paul J, Brito BR, Ruan Y, Swan BK, Valentine RSDL, Thurber RV, Wegley L, White BA, Rohwer F (2008). Functional metagenomic profiling of nine biomes. Nature.

